# Protocol for analysis of miRNAs in human melanoma cells

**DOI:** 10.1016/j.xpro.2025.103861

**Published:** 2025-05-30

**Authors:** Evelyn Lattmann, Tessa Guggiari, Patrick Turko, Mitchell P. Levesque

**Affiliations:** 1 Department of Dermatology, University of Zurich, University Hospital Zurich, 8952 Schlieren, Switzerland

**Keywords:** Cell Biology, Cell culture, Cell-based Assays, Cancer, Microscopy, In Situ Hybridization

## Abstract

MicroRNAs (miRNAs) significantly contribute to melanoma plasticity, a key factor in metastasis and therapy resistance. Here, we provide a protocol for analysis of miRNAs in human melanoma cells. We describe two approaches for plating cells, either using cell chambers or attaching cells to microscopy slides by means of centrifugation. We first focus on cell pretreatment and staining procedures using the RNAscope Plus small RNA-RNA high-definition (RNAscope Plus smRNA-RNA HD) assay. We then detail the quantitative analysis of miRNA expression and co-localization.

## Highlights

• Steps for co-detection of microRNAs and mRNAs in melanoma cells
• Instructions for preparing adherent and suspension cells for microRNA/mRNA co-staining
• Guide to image acquisition, segmentation, and analysis using Vectra Polaris and QuPath

## Abstract (editor highlights)

Publisher’s note: Undertaking any experimental protocol requires adherence to local institutional guidelines for laboratory safety and ethics.

## Graphical abstract

**Figure:**
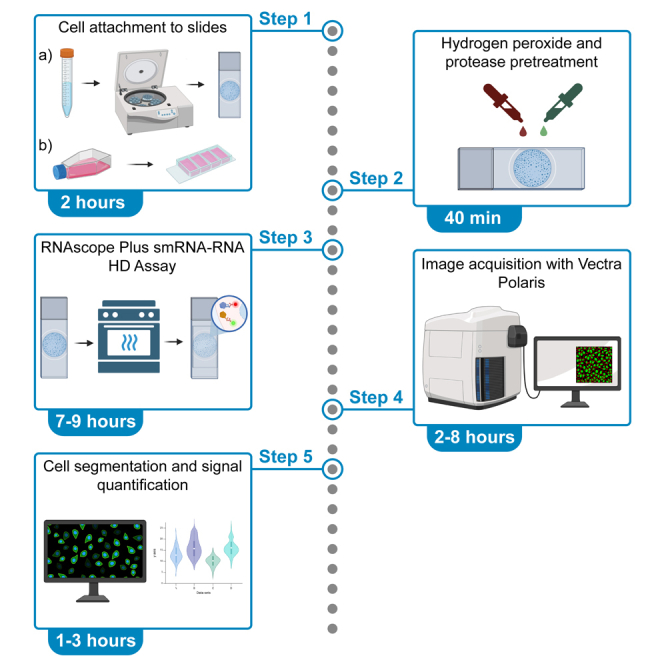

## Before you begin

miRNAs are crucial regulators of gene expression and have been linked to the pathogenesis of melanoma, a malignant skin cancer originating from melanocytes.^1^^,^^2^ miRNAs can function as oncogenes (oncomiRs) or tumor suppressors, influencing melanoma development, progression, and therapy resistance.^3^^,^^4^ Techniques such as in situ hybridization enable the detailed spatial examination of miRNAs within single cells and tissue samples, enhancing our understanding of their roles in melanoma. The outlined protocol details the specific steps for spatial miRNA analysis in melanoma cells using RNAscope Plus. Additionally, we have successfully employed this protocol with suspension cells such as peripheral blood mononuclear cells (PBMCs), THP-1 and NK cells. While the protocol is adaptable to various cell types, pretreatment conditions may require optimization, as elucidated in the provided protocol.

### Institutional permissions

The study is conducted under the ethical approval of BASEC Nr. 2017-00494 and BASEC Nr. 2014-0425, adhering to all ethical standards for research involving human participants. Before proceeding, ensure compliance with regulatory standards and obtain the necessary authorizations to work with human cell lines from the relevant institutions.

### Preparation of Roswell Park Memorial Institute cell culture medium

**Timing: 10 min**

1. Prepare RPMI cell culture medium for maintaining and collecting cell lines. It can be prepared in advance and stored at 4°C for up to 2 months (For details see materials and equipment setup):
  a. Add 1% sodium pyruvate (1 mM), 2% L-glutamine (4 mM), and 10% heat-inactivated fetal bovine serum (FBS) to the RPMI-1640 medium.
  b. Mix well and sterile filter the RPMI cell culture medium with a polyethersulfone (PES) membrane filter (0.22 μm) using a vacuum pump.

***Note:*** No antibiotics were included in the RPMI cell culture medium. Mycoplasma contamination was monitored using PlasmoTest; however, other testing methods can also be employed.

### Cell seeding into cell culture flasks or 4-well culture slides

**Timing: 45 min–1 h**

2. Seed cells one day prior to the assay to achieve optimal confluency:
  a. **For cell culture flasks (preparation for Step 1a):** seed cells into a T75 cell culture flask to achieve approximately 80% confluency the next day.
  b. **For 4-well culture slides (preparation for Step 1b):** Plate 200,000 cells per chamber of a 4-well culture slide for every cell line.
3. Aspirate the medium and wash cells with 1x PBS.
4. Detach cells using 1 mL 0.05% Trypsin-EDTA in a T75 cell culture flask.
5. Resuspend cells in 5 mL of RPMI cell culture medium and transfer the suspension to a 15 mL centrifuge tube.
6. Centrifuge the 15 mL centrifuge tube at 300 g for 3 min at 22°C.
7. Aspirate the supernatant and resuspend the cell pellet in 2 mL of medium.
8. Count cells using a cell counter or manually.
  a. **For cell culture flasks (preparation for Step 1a):** Plate 1/4 to 1/2 of the cells into a fresh T75 cell culture flask.
  b. **For 4-well culture slides (preparation for Step 1b):** plate 200,000 cells per 4-well culture slide. Fill each chamber of the 4-well culture slide with RPMI cell culture medium to a final volume of 800 μL.

### Preparation of fresh 4% paraformaldehyde

**Timing: 5 min**

4% PFA will be used in Step 1a and Step 1b to fix cells and preserve their architecture.

***Note:*** Unused 4% PFA can be stored at −20°C for a few months. But for best results 4% PFA should be prepared just before use.

9. Carefully break open the glass ampule containing 16% PFA by removing the upper part, using a clean tissue to protect your fingers from cuts.
10. Transfer the 16% PFA to a 15 mL centrifuge tube by pipetting it from the broken ampule.
11. In a fresh 15 mL centrifuge tube pipette 1 volume of 16% PFA into 3 volumes of PBS (4x dilution). For example, 1 mL 16% PFA in 3 mL PBS is enough for 10 cell line samples (c.a. 400 μL are needed per cell line sample).
12. Use immediately for fixation in (Step 1a and Step 1b).

### Warm up the HybEZ II hybridization system

**Timing: 35 min**

The HybEZ II Hybridization System provides a controlled temperature and humidity environment to ensure optimal and consistent hybridization conditions during the RNAscope assay.

13. Place a Humidifying Paper into the Humidity Control Tray and thoroughly wet it with distilled water.
14. Insert the Humidity Control Tray into the HybEZ II Hybridization System.
15. Warm up the HybEZ II Hybridization System at 25°C (Hold RT) for 30 min before protease III treatment.

***Note:*** After completing the protease treatment, warm up the HybEZ II Hybridization System to 40°C for 10 min before starting the RNAscope assay. Always keep the Humidity Control Tray in the HybEZ II Hybridization System when not in use.

### Preparation of target probe mix

**Timing: 5–10 min**

This protocol allows for the simultaneous visualization up to three mRNAs and one miRNA. To prepare the probe mix, the S1 miRNA probe is used as a diluent for combining probes C2, C3, and C4. If miRNA detection is not required, the Probe Diluent (instead of S1, see key resources table for details) can be used to dilute the mRNA probes.

***Note:*** Either the S1 miRNA probe or the Probe Diluent must be used as a base for the mixture, but Probes C2, C3, and C4 can be added in any combination, depending on the target mRNAs of interest.

16. Briefly spin down the RNAscope Probes C2, C3, and C4.

***Note:*** Approximately 50–100 μL of target probe mix is required per cell line slide. If processing multiple slides with the same probe set, a master mix can be prepared.

17. To measure accurate amounts of S1 (supplied in a squeeze bottle), add a few drops—equivalent to the volume required for the dilution—into a 1.5 mL microcentrifuge tube. For example, 7–10 drops (approximately 100 μL) are needed to prepare the target probe mix for one cell culture slide.
18. Pipette 50 volumes of S1 and 1 volume each of C2, C3, and C4 RNAscope Probes (50:1:1:1 ratio) into a new clean 1.5 mL centrifuge tube. Mix well by gently inverting the tube. If using the C2, C3, or C4 RNAscope Probes separately, dilute them with Probe Diluent. For example, to prepare the target probe mix for one cell culture slide:
  a. S1 Probe: 100 μL.
  b. C2, C3, C4 Probes: 2 μL each.

***Note:*** The mixed RNAscope Probes can be stored at 2°C–8°C for up to six months.

### Preparation of tyramide signal amplification Vivid/Opal fluorophores

**Timing: 5–10 min**

Select the appropriate TSA Vivid/Opal fluorophores recommended in the RNAscope Plus smRNA-RNA Assay protocol. Prepare only the fluorophores that you will use for the assay. You can combine RNAscope Probes and TSA Vivid/Opal fluorophores as needed.

19. Dilute the TSA Vivid/Opal fluorophores in a range of 1:750 to 1:3000 in TSA Buffer provided in the RNAscope Plus HD Reagent Kit.

***Note:*** 1:1500 dilution of each TSA Vivid fluorophore was used in our assay.

### Preparation of 1x RNAscope wash buffer

**Timing: 5–10 min**

For the assay, prepare 3 L of 1x RNAscope Wash Buffer that is supplied with the RNAscope Plus smRNA-RNA HD Reagent Kit (see details in key resources table).

20. Add 60 mL (1 bottle) of 50x RNAscope Wash Buffer into 2.94 L of distilled water.
21. Mix well to ensure thorough dilution.

***Note:*** If precipitation occurs in the 50x Wash Buffer bottle, warm it at 40°C for 10–20 min before use. The prepared 1x RNAscope Wash Buffer can be stored at 20°C–25°C for up to one month.

### Equilibration of reagents at 20°C–25°C before use

**Timing: 30–45 min**

22. Allow the following smRNA-RNA HD reagents to equilibrate to 20°C–25°C before use: AMP1, AMP2, AMP3, HRP-S1, HRP-C2, HRP-C3, HRP-C4, and HRP-blocker.

## Key resources table

**Table:** 

REAGENT or RESOURCE	SOURCE	IDENTIFIER
**Chemicals, peptides, and recombinant proteins**		

RPMI 1640	Sigma-Aldrich	R0883
Heat-inactivated fetal bovine serum (FBS)	Biowest	S1810
L-glutamine (200 mM)	Gibco	2503024
Sodium pyruvate (100 mM)	Sigma-Aldrich	S8636
Phosphate-buffered saline (PBS)	Gibco	10010015
0.05% trypsin-EDTA	Gibco	250300054
16% paraformaldehyde (PFA)	Thermo Fisher Scientific	043368.9M
PlasmoTest	InvivoGen	rep-pt1
Anti-fade fluorescence mounting medium	Abcam	ab104135
Ethanol	Sigma-Aldrich	51976

**Critical commercial assays**		

RNAscope Plus smRNA-RNA HD reagent kit (contains the RNAscope Plus smRNA-RNA HD detection kit including DAPI, RNAscope H202 and protease reagents, RNAscope target retrieval reagents, RNAscope wash buffer, and TSA buffer)	Bio-Techne	322785
RNAscope Plus smRNA-RNA HD detection kit^a^	Bio-Techne	32780
RNAscope H202 and protease reagents (contains hydrogen peroxide solution, protease III)	Bio-Techne	322381
RNAscope target retrieval reagents	Bio-Techne	322000
RNAscope wash buffer^a^	Bio-Techne	310091
TSA buffer^a^	Bio-Techne	322809
RNAscope DAPI^a^	Bio-Techne	320858
miRNA probe, e.g., MIMAT0000449	Bio-Techne	887771-S1
mRNA probe C2, e.g., RNAscope probe - Hs-MITF-C2	Bio-Techne	310951-C2
mRNA probe C3, e.g., RNAscope probe - Hs-BGN-C3	Bio-Techne	415491-C3
RNAscope probe diluent	Bio-Techne	300041
RNAscope Plus smRNA-RNA 4-plex negative control probe	Bio-Techne	323391
RNAscope Plus smRNA-RNA 4-plex positive control probe	Bio-Techne	323361
TSA Vivid Fluorophore 650	Bio-Techne	323273
TSA Vivid Fluorophore 570	Bio-Techne	323272
TSA Vivid Fluorophore 520	Bio-Techne	323271

**Experimental models: Cell lines**		

Human: melanoma cell line M130219	Biobank, USZ^5^	N/A
Human: melanoma cell line M130429	Biobank, USZ^5^	N/A

**Software and algorithms**		

Vectra Polaris version 1.0.13	Akoya Biosciences	N/A
Phenochart version 1.2.0	Akoya Biosciences	N/A
QuPath version 0.4.3	N/A	N/A
RStudio version 2022.12.0+353	N/A	N/A

**Other**		

Vacuum filtration 0.2 μm PES membrane filter	TPP	99500
SuperFrost Plus slides	Faust	9.161 155
4-well culture slides	Falcon	354114
15 mL centrifuge tube	TPP	91015
1.5 mL centrifuge tube	Eppendorf	0030120086
Sorvall Heraeus swinging bucket centrifuge rotor	Thermo Fisher Scientific	75006445
Heraeus Multifuge 3s centrifuge	Thermo Fisher Scientific	N/A
Funnel clip	Simport Scientific	SKU M964B
Cytospin funnel (TPX single sample chamber)	Thermo Fisher Scientific	A78710018
White filter paper (for TPX funnel)	Thermo Fisher Scientific	M965FWDV
Cytospin III centrifuge	Thermo Fisher Scientific Shandon	N/A
PhenoImager HT (formerly Vectra Polaris)	Akoya Biosciences	N/A
ImmEdge hydrophobic barrier pen	Vector Laboratories	H-4000
Tissue-Tek slide container	Tissue-Tek	62541-01
Tissue-Tek slide holder	Tissue-Tek	62543-06
HybEZ II hybridization system	ACD	321711 or 321721
HybEZ humidity control tray (with lid)	ACD	310012
EZ-Batch slide holder (20 slide capacity)	ACD	321716
EZ-Batch wash tray	ACD	321717
HybEZ humidifying paper	ACD	310015

a Listed reagents are contained in the RNAscope Plus smRNA-RNA HD Reagent Kit but are also available for individual purchase.

## Materials and equipment

**Table:** RPMI cell culture medium

Reagent	Final concentration	Amount
RPMI-1640	N/A	435 mL
Sodium pyruvate (100 mM)	1 mM (1%)	5 mL
L-glutamine (200 mM)	4 mM (2%)	10 mL
Heat-inactivated fetal bovine serum (FBS)	10%	50 mL
**Total**	**N/A**	**500 mL**

Sterile filter the RPMI cell culture medium with a PES membrane filter (0.22 μm) and store at 4°C for up to 2 months.

***Note:*** For convenience, we do not remove 65 mL from the purchased 500 mL RPMI-1640 medium bottle. Regardless of the approach, the medium should be prepared in a consistent manner for all experiments.

**Critical reagents:** The RNAscope Plus smRNA-RNA HD Reagent Kit is required for the completion of this protocol.

***Alternatives:*** Step 1a of the protocol uses a Cytospin centrifuge of the model Thermo Shandon Cytospin III. However, other Cytospin centrifuge models may be used. When using an alternative device, make sure that the Funnel Clip, the Cytospin funnel and White Filter Papers are compatible with the device. If cells are adherent, 4-well culture slides can be used instead of depositing cells onto slides (see Step 1b). PBMCs may be plated by doing a "blood-smear".

***Alternatives:*** In Step 3 a HybEZ II Hybridization System for the incubation steps. A hand-made humidity chamber can be placed into a standard incubator if no HybEZ II Hybridization System is available.

***Alternatives:*** Step 4 uses the PhenoImager HT (formerly Vectra Polaris) from Akoya for imaging slides. This system is equipped with a 20x/0.75 NA objective and supports digital magnification up to 40x. The PhenoImager HT utilizes the following LED excitation wavelengths: UV (380–390 nm) / Violet (421–439 nm) / Blue (464–486 nm) / Yellow (509-591 nm) / Red (629–647 nm) / NIR (719-751 nm); and the following fluorescent band filters: DAPI (Hoechst), OPAL520 (FITC, Alexa 488, GFP), OPAL570 (Alexa 555, RFP, tdTomato), OPAL690 (Alexa 647, Cy5), OPAL480, OPAL620 (Alexa 594, Alexa 568) and OPAL780. Other slide scanners (e.g., Zeiss Axioscan, Leica Cell Dive) or confocal microscopes may be used for fluorescence signal detection.

***Alternatives:*** Step 5 uses the Image processing software QuPath version 0.4.3 for cell segmentation and signal quantification. Other image processing software (e.g., ImageJ (Fiji)) may be used, but require adaptations of the protocol.

## Step-by-step method details

The first part of this section outlines the procedure for fixing cells onto imaging slides and the pretreatment steps required to ensure optimal visualization of mRNAs and miRNAs. The subsequent RNAscope Plus smRNA-RNA HD Assay (developed by ACD) is not covered in detail here, as it is fully optimized, and a user manual is available directly from the company. To perform this assay, we recommend contacting ACD to obtain the user manual. Visit the following link to initiate this process: https://acdbio.com/rnascopeplus. The second part of this section describes the specific steps for imaging slides using the Vectra Polaris system, provides an overview of the expected results, and offers recommendations for data quantification and visualization.

### Cell attachment to slides

**Timing: 2 h**

Cells can be attached to slides either by culturing them on plates or in suspension and subsequently depositing them onto slides using a Cytospin centrifuge (Step 1a) or by growing them directly in commercially available culture slides (Step 1b).

1. Attach cells to onto Superfrost Plus slides by using a Cytospin centrifuge (Step 1a).
  **CRITICAL:** Before initiating the assay, it is essential to attach the cells onto Superfrost Plus slides to preserve cellular architecture and protect against RNA degradation. The detailed steps are outlined below. For each slide, you will need a final concentration of 100,000 cells per 100 μL (equivalent to 1 × 10^6^/mL) in 1x PBS.
  a. Aspirate medium from the T75 cell culture flask.
  b. Detach cells using 1 mL 0.05% Trypsin-EDTA.
  c. Resuspend cells in 4 mL of RPMI cell culture medium and transfer the cell suspension into a 15 mL centrifuge tube.
  d. Centrifuge the 15 mL centrifuge tube at 300 g for 5 min at 4°C.
  e. Gently aspirate off supernatant.
  f. Wash the cell pellet once with 5 mL 1x PBS.
  g. Centrifuge the 15 mL centrifuge tube again at 300 g for 5 min at 4°C.
  h. Gently aspirate the 1x PBS.
  i. Add 400 μL of freshly prepared 4% PFA to the cell pellet and mix gently by pipetting up and down.
    **CRITICAL:** PFA is toxic. Handle in a fume hood while wearing appropriate personal protective equipment. Dispose of waste according to institutional safety guidelines.
  j. Incubate for 10 min at 20°C–25°C.
  k. Gently remove 4% PFA and dispose of it according to the biosafety and waste disposal guidelines.
  l. Wash cells three times with 500 μL 1x PBS:
    i. Resuspend the cells in 500 μL 1x PBS.
    ii. Centrifuge at 300 g for 2 min.
    iii. Aspirate the 1x PBS.
    iv. Repeat steps (a–c) two more times.
  m. Resuspend the cells to a concentration of 100,000 cells per 100 μL (= 1 × 106/mL) in 1x PBS.
  n. Let the cells stand in 1x PBS while assembling the Cytospin III centrifuge:
    i. Place the Superfrost Plus slide into the Funnel Clip (Figure 1), with the top of the slide facing upward.

**Figure 1:**
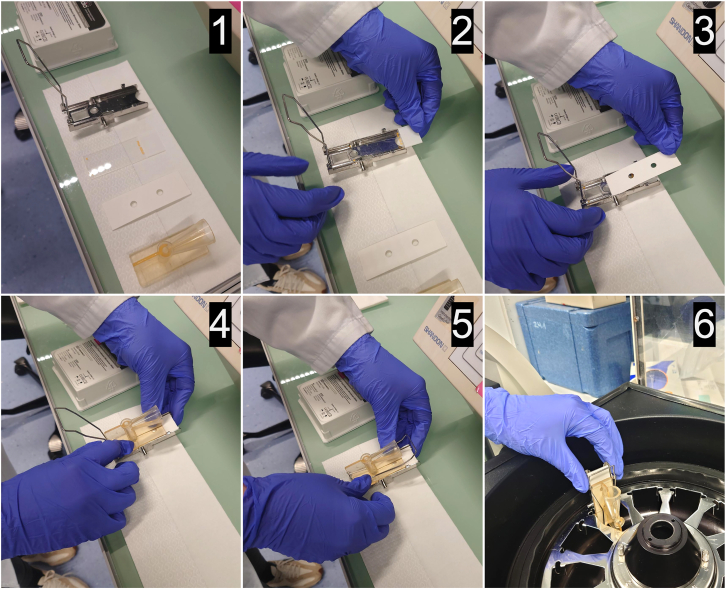
Preparation of the Cytospin assembly The figure illustrates the steps (1–6) to prepare the cytospin assembly. (1) Confirm the presence of all required components: Funnel Clip, White Filter Paper, Superfrost Plus slide, and Cytospin funnel. (2) Position the Superfrost Plus slide onto the Funnel Clip, with the top of the slide facing upward. (3) Place the White Filter Paper on top of the slide. (4) Carefully position the Cytospin funnel onto the filter. (5) Close the metal clip to secure all components in place. (6) Insert the assembled sample chamber into the Cytospin centrifuge rotor, ready for cell deposition onto the slide.
    ii. Place a White Filter Paper above the Superfrost Plus slide.
    iii. Finally, place the Cytospin funnel onto the filter.
    iv. Secure the assembly by locking the metal clip into the hooks.
  o. Place the assembly into the Cytospin III centrifuge.
  p. Load 100 μL of the cell suspension into the Cytospin funnel.
  q. Centrifuge at 800 rpm for 5 min.
  r. Carefully remove the assembly and disassemble the Cytospin funnel and White Filter Papers.
  s. Draw a thin border around the cell spot using the ImmEdge Hydrophobic Barrier Pen and let it dry for 5 min.
    **CRITICAL:** Do not let the cells dry out during this process. After 2 min, you may add a small drop of 1x PBS onto the center of the cell spot, allowing it to spread evenly to the ImmEdge Hydrophobic Barrier Pen border by the end of the 5 min.
2. Attach cells using 4-well culture slides (Step 1b).
  **CRITICAL:** For adherent cells, there is the possibility to directly culture cells on microscopy slides using 4-well culture slides, where the upper plastic chamber will be removed before imaging. Like for cytospinned cells, cells must be fixed before the staining.
  a. Gently aspirate the RPMI cell culture medium from the chambers of the 4-well culture slides.
  b. Wash the cells once with 400 μL 1x PBS: Pipette the PBS carefully along the border of the chambers to avoid detaching the cells.
  c. Add 400 μL of freshly prepared 4% PFA to each chamber and incubate at 20°C–25°C for 30 min (see troubleshooting problem 1).
  d. Gently remove the 4% PFA and dispose of it according to the biosafety and waste disposal guidelines.
  e. Wash the cells three times with 200 μL of 1x PBS per chamber:
    i. Carefully add 200 μL 1x PBS per chamber.
    ii. Remove 1x PBS.
    iii. Repeat steps (a) and (b) two more times.
3. Optional: Dehydrate the cells for storage (Step 1a and Step1b).
  **Pause Point:** If you wish to preserve your fixed cells on the Superfrost Plus slide without immediately proceeding to the RNAscope Plus smRNA-RNA HD Assay, you can dehydrate the cells and store them in 100% ethanol at −20°C for up to six months.
  a. Submerge slides in a Tissue Tek slide container filled with 50% ethanol for 1 min at 20°C–25°C.
  b. Transfer the slides directly to a Tissue Tek slide container filled with 70% ethanol for 1 min at 20°C–25°C.
  c. Transfer the slides directly to a Tissue Tek slide container filled with 100% ethanol for 1 min at 20°C–25°C.
  d. Replace the 100% ethanol with fresh 100% ethanol and store the slides in it at −20°C for up to six months.
4. Optional: Rehydrate the cells after storage (Step 1a and Step 1b).
  **CRITICAL:** Prior to proceeding with the RNAscope Plus smRNA-RNA HD Assay after storage, it is necessary to rehydrate the cells.
  a. Submerge the slides in a Tissue Tek slide container filled with 100% ethanol for 1 min at 20°C–25°C.
  b. Transfer the slides directly to a Tissue Tek slide container filled with 70% ethanol for 1 min at 20°C–25°C.
  c. Transfer the slides directly to a Tissue Tek slide container filled with 50% ethanol for 1 min at 20°C–25°C.
  d. Replace the 50% ethanol with fresh 1x PBS and incubate the slides for 10 min at 20°C–25°C.
  e. Proceed directly to the hydrogen peroxide pretreatment step.

### Hydrogen peroxide and protease pretreatment

**Timing: 40 min**

To block the activity of endogenous peroxidase, a pretreatment with hydrogen peroxide is performed. The following sections outline hydrogen peroxide and protease pretreatment steps for either cytospin-deposited cells (Step 2a) or cells cultured in 4-well slides (Step 2b).

5. Perform hydrogen peroxide and protease treatment of cells plated by Cytospin (Step 2a).
  a. Place the slides on a clean tissue on the benchtop.
  b. Add 1–2 drops of Hydrogen Peroxide solution onto the cell spot.
  c. Incubate for 10 min at 20°C–25°C.
  d. Place slides into a Tissue Tek slide holder and submerge them in 1x PBS in a tissue staining dish (e.g., Tissue Tek slide container) to wash the slides.
  e. Repeat the wash with fresh 1x PBS.
  f. Proceed immediately to the protease treatment.
    ***Note:*** Optimize for the appropriate protease dilutions depending on the cell lines. A 1:15 protease typically results in the highest intensity-to-noise ratio for most of the cell lines (see troubleshootingproblem 2).
  g. Dilute protease in 1x PBS choosing from the following options:
    i. 1:7.5 (4 μL protease III in total volume of 30 μL per sample).
    ii. 1:15 (2 μL protease III in total volume of 30 μL per sample).
    iii. 1:30 (1 μL protease III in total volume of 30 μL per sample).
  h. For each slide add 30 μL of the diluted Protease III onto the cell spot.
  i. Arrange the slides into the EZ-Batch Slide Holder and place it into the HybEZ II Hybridization System.
  j. Incubate the slides in the HybEZ II Hybridization System at 25°C (Hold RT) for 10 min.
  k. Take out the EZ-Batch Slide Holder and place it into the EZ-Batch Wash Tray filled with 1x PBS.
  l. Wash slides for 2 min in 1x PBS.
  m. Repeat the wash with fresh 1x PBS.
  n. Proceed directly to the RNAscope Plus smRNA-RNA HD Assay.
6. Perform hydrogen peroxide treatment for cells in 4-well culture slides (Step 2b).
  a. Add 2 drops of Hydrogen Peroxide solution into each compartment of the 4-well culture slide and incubate for 10 min at 20°C–25°C.
  b. Wash once with 1x PBS by adding 800 μL 1x PBS into each chamber.
  c. Repeat the wash with fresh 1x PBS.
  d. Remove the chamber using the appropriate removal device (provided with the slides):
    i. Position the slide within the device.
    ii. Carefully slide the white piece between the chambers and the slide.
    iii. Detach the chambers carefully.
  e. Submerge the unmounted slides in a tissue staining dish (e.g., Tissue Tek slide container) filled with 1x PBS.
  f. Draw a thin border around the cell area with the ImmEdge Hydrophobic Barrier Pen and allow it to dry for 5 min.
  g. Place the slides on a clean tissue on the benchtop.
  h. Add 2–3 drops of Hydrogen Peroxide solution onto the cell area.
  i. Incubate for 10 min at 20°C–25°C.
  j. Place slides into a Tissue Tek slide holder and submerge them in 1x PBS in a tissue staining dish (e.g., Tissue Tek slide container) to wash the slides.
  k. Repeat the wash with fresh 1x PBS.
  l. Proceed immediately to the protease treatment.
    ***Note:*** Optimize for the appropriate protease dilutions depending on the cell lines. A 1:15 protease typically results in the highest intensity-to-noise ratio for most of the cell lines (see troubleshootingproblem 2).
  m. Dilute protease in 1x PBS choosing from the following options:
    i. 1:7.5 (4 μL protease III in total volume of 30 μL per sample).
    ii. 1:15 (2 μL protease III in total volume of 30 μL per sample).
    iii. 1:30 (1 μL protease III in total volume of 30 μL per sample).
  n. For each slide add 50 μL of diluted Protease III onto the cell area.
  o. Arrange the slides into the EZ-Batch Slide Holder and place it into the HybEZ II Hybridization System.
  p. Incubate the slides in the HybEZ II Hybridization System at 25°C (Hold RT) for 10 min.
  q. Take out the EZ-Batch Slide Holder and place it into the EZ-Batch Wash Tray filled with 1x PBS.
  r. Wash slides for 2 min in 1x PBS.
  s. Repeat the wash with fresh 1x PBS.
  t. Proceed directly to the RNAscope Plus smRNA-RNA HD Assay.
    **CRITICAL:** Do not let the cells dry out at any point during this process.

### RNAscope plus smRNA-RNA HD assay

**Timing: 7–9 h**

The initial step involves the RNAscope Probes hybridizing with the target RNA for a duration of 2 hours. This is followed by signal amplification through multiple phases: pre-amplifiers bind specifically to the hybridized RNAscope Probes, and amplifiers attach to the pre-amplifiers, enabling the binding of a labeled probe conjugated with HRP. The HRP enzyme activates fluorophore-labeled tyramide, which reacts with protein tyrosine residues to form a covalent bond.

7. To perform the RNAscope Plus smRNA-RNA HD Assay, follow the manual provided by ACD. Make sure to distribute reagents evenly over the cells (see troubleshooting problem 3). We followed the protocol exactly as indicated in the manual. For the TSA Vivid fluorophores, we used a dilution of 1:1500 (see “before you begin” section).

***Note:*** Use 30 or 50 μL of TSA Vivid/Opal fluorophores per cell spot or cell area, respectively.

**CRITICAL:** Ensure thorough washing of the probes and TSA Vivid/Opal fluorophores to minimize background noise and non-specific signal. Gently agitate the Wash buffer over the slides by using slow rocking motions with the EZ-Batch Wash Tray. The cells should adhere firmly enough to remain attached throughout the washing process.

8. Counterstain and mount the slides.
  a. After the final wash, remove excess liquid from the slide.
  b. Add 1 drop of DAPI to cover the cell spot or cell area.
  c. Incubate for 30 s to 5 min at 20°C–25°C. In our hands 5 min incubation time worked best (A longer incubation period of 5 min may be needed for optimal cell segmentation).
  d. Remove the DAPI by gently tapping the slide onto an absorbent paper.
  e. Briefly wash the slide in distilled water.
  f. Immediately add 1–2 drops of Anti-Fade Fluorescence Mounting Medium onto each slide.
  g. Cover each slide with a 24 × 50 mm coverslip, avoiding the trapping air bubbles under the coverslip.
    ***Note:*** If air bubbles become trapped under the coverslip, carefully use the tip of a closed pair of tweezers to apply gentle pressure near the air bubble, directing it towards the edge of the slide. Perform this action delicately to avoid shifting the coverslip.
  h. Allow the slides to dry in the dark at 20°C–25°C for 30 min to 2 h.
  i. Store slides at 4°C overnight in the dark.
    **CRITICAL:** Let the mounting medium dry at least for 8 h before the imaging.
    ***Note:*** Due to the intense brightness of the TSA Vivid fluorophores' signal, imaging remains viable for up to two weeks post-procedure. For optimal results, perform imaging within the initial 1–5 days after completing the assay.

### Image acquisition with Vectra Polaris

**Timing: 2–8 h (depending on the number of slides, RNAscope probes, exposure time, and cell spot area)**

This step involves imaging of slides with PhenoImager HT (formerly Vectra Polaris). For a reliable comparison among samples, maintaining uniform acquisition times is critical.

9. Clean the slides with 70% Ethanol and a clean tissue before imaging.
10. Use a black pen to draw a circle on the coverslip to ensure that the ImmEdge Hydrophobic Barrier Pen border is fully covered.
11. Insert slides into a Vectra Polaris Rack.

**CRITICAL:** Make sure the ImmEdge Hydrophobic Barrier Pen border is fully covered. Incomplete coverage may interfere with slide scanning.

**Figure 2:**
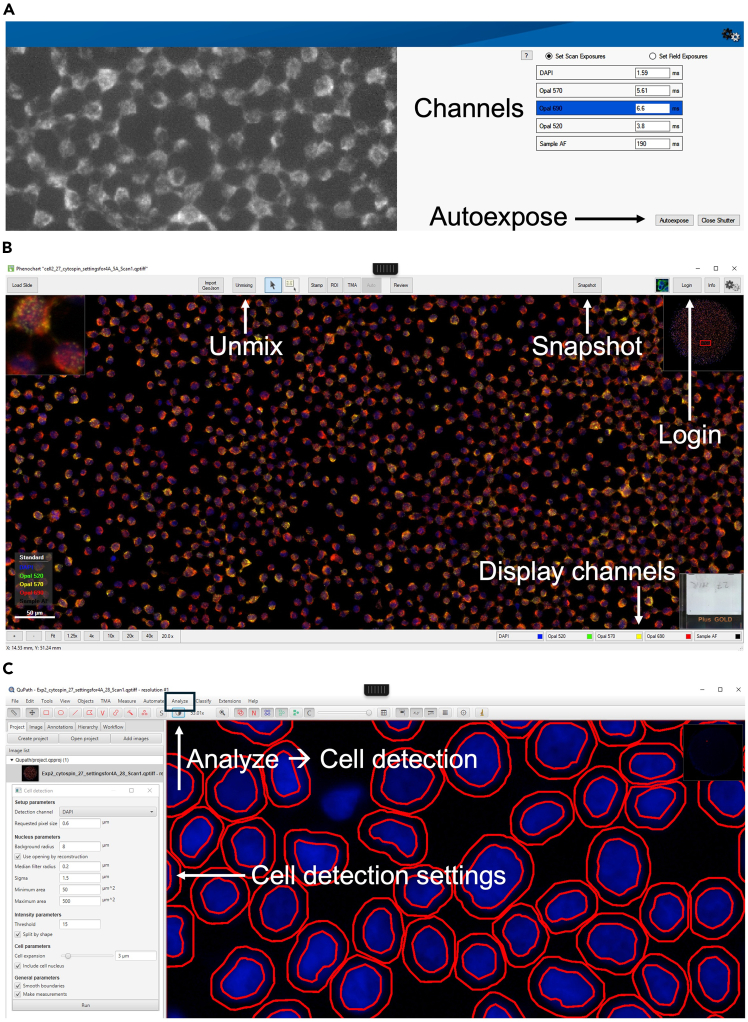
Image acquisition and cell segmentation (A) Overview of the acquisition interface of the Vectra Polaris software with the channel settings used for acquisition. (B) Screenshot of the Phenochart interface to visualize the acquired images. (C) Overview of the QuPath interface used for cell segmentation and fluorescence intensity quantification.

12. Open Vectra Polaris software 1.0.13.
13. Click on **Edit Exposure**, then select **New.**
14. Enter a protocol name and select **Fluorescence.** Configure the required settings for imaging:
  a. **Overview Scan Filter**: Select DAPI.
  b. **Pixel Resolution:** Set to 40x.
  c. **Filters:** Choose the required filters by clicking on **Select Scan Bands**.
15. Click on **Scan exposure** to set the exposure time of each channel.
16. Click on **Load Carrier** and select the carrier containing the slide.
17. Select **Take Overview** to acquire an overview image of the slides.
18. Click on the cell spot and select **Auto Focus** using the channel detecting the miRNA (*It is recommended to focus on the miRNA signal instead of the nucleus signal*).
19. If the focus is significantly off, use the focus adjustment bar located below the **Auto Focus** button to fine-tune the focus.
20. Adjust the exposure time for each channel for every sample based on the highest fluorescence intensity using the **Auto Exposure** button (Figure 2A).

**CRITICAL:** Examine the signal intensity across all samples to identify the one with the highest fluorescence intensity. Adjust the exposure time for each channel based on this sample. This ensures optimal imaging conditions tailored to the most intense signals and allows for consistent comparisons across all samples.

21. Click on Back and Save, to save the protocol.
22. Click on **Back** again to return to the Home page.
23. On the Home page select **Scan Slide.**
24. Click on **Configure Task.** A new window will open:
  a. Task: Select **Scan slides**.
  b. Select a Study and a Protocol.
  c. Enter a **Slide ID**.
25. Click **Scan** to start the whole slide scanning.

### Cell segmentation and signal quantification

**Timing: 1–3 h**

This step involves segmenting each cell line to quantify the fluorescence intensity for each channel at the single-cell level and to enable further analysis.

#### Visualizing data in Phenochart 1.2.0

26. Open Phenochart.
27. Open the Akoya whole slide scan file (.qptiff) generated during the imaging process in Phenochart.
28. Click on **Login** and enter a username (Figure 2B).
29. Click on **Unmixing** and select **Opal + AF.**
30. To visualize individual channels, deselect all channels except the one you want to view.
31. Click on **Snapshot** to acquire snapshots if needed.

#### Cell segmentation in QuPath 0.4.3

32. Open QuPath 0.4.3.
33. Click on **File** – **Project** – **Create project**.
34. Click on **Add images:**
  a. Image provider: Default (let QuPath decide).
  b. Set image type: Fluorescence.
  c. Rotate image: Leave empty.
  d. Click on **choose files** and select the scanned image (.qptiff file).
  e. Click on **Import.**
35. To define a region of interest (ROI) click on Annotations and draw a circle around the cell spot.
36. Perform cell segmentation: Click on **Analyze** – **Cell detection** – **Cell detection** (Figure 2C). A settings window will open. Use the following parameters to segment melanoma cells (adjust as needed for optimal segmentation):
  a. Requested pixel size: 0.6 μm.
  b. Background radius: 8 μm.
  c. Median filter radius: 0.2 μm.
  d. Sigma: 1.5 μm.
  e. Minimum area: 50 μm^2^
  f. Maximum area: 500 μm^2^
  g. Threshold: 15
  h. Cell expansion: 3 μm.
  i. Check all boxes.

**CRITICAL:** Ensure that the expansion parameter is not excessively large so that cell segmentations of neighboring cells do not overlap. Overlapping can result in inaccurate signal measurements due signal spill-over.

37. Select **Measure** – **Show detection measurements** to open a table displaying the detection measurements.
38. Click on **Save**, to export the table for further data processing and analysis.
39. Visualize miRNA and mRNA quantifications with Rstudio: For the visualization of single-cell fluorescence intensity data, we suggest violin plots, as they effectively display the distribution of fluorescence intensity across cells. Below is the R code to generate the graphs with a data frame of the following structure (Column 1 = cell_line (e.g., “cell_line_1”, “cell_line_2”; Column 2 = fluorescence_intensity (e.g., 17.024, 20.928); Column 3 = fluorophore (e.g., fluorophore_1).

# Load necessary libraries

library(ggplot2)

library(scales)

# Plot fluorescence intensity across fluorophores for two cell lines

ggplot(df, aes(x = cell_line, y = fluorescence_intensity, fill = cell_line)) +

 geom_violin() + # Violin plot

 geom_boxplot(fill = "snow", outlier.shape = NA, width = 0.2) + # Boxplot overlay

 scale_fill_manual(values = c("cell_line_1" = "blue", "cell_line_2" = "orange")) + facet_wrap(∼fluorophore, scales = "free_y", ncol = 3, nrow = 1) + scale_y_log10(labels = trans_format("log10", math_format(10ˆ.x))) + # Log scale ylab("Fluorescence Intensity") +

 xlab("") +

 ggtitle("Your title") +

 theme(

  plot.title = element_text(size = 12, hjust = 0.5),

  axis.text.x = element_text(size = 13, angle = 45, vjust = 0.5),

  axis.text.y = element_text(size = 10),

  strip.text = element_text(size = 9),

  axis.title.y = element_text(size = 13),

  legend.position = "right",

  strip.background = element_blank(),

  panel.grid.minor.y = element_blank(),

  panel.grid.major.y = element_line(colour = "grey85", linewidth = 0.1),

  panel.background = element_blank(),

  panel.grid.major.x = element_line(colour = "grey85", linewidth = 0.1),

  axis.line.x = element_line(linewidth = 0.25),

  axis.line.y = element_line(linewidth = 0.25))

### Biosafety instructions and waste removal

Always handle PFA (paraformaldehyde) within a fume hood while wearing appropriate personal protective equipment (PPE), including gloves and safety glasses. Dispose PFA solutions exclusively in chemical hazardous waste containers.

## Expected outcomes

**Figure 3:**
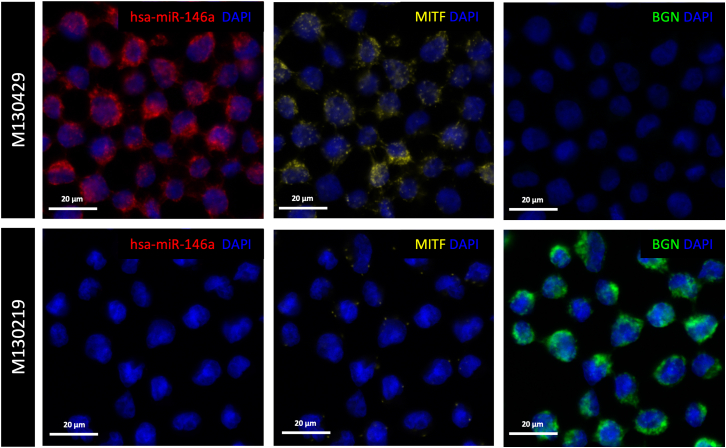
Spatial localization of hsa-miR-146a-5p, MITF, and BGN in melanocytic and mesenchymal melanoma cells Visualization of hsa-miR-146a-5p (left panel, red), *MITF* mRNA (middle panel, yellow) and *BGN* (right panel, green) in melanocytic melanoma cells (M130429, upper row) and in mesenchymal melanoma cells (M130219, lower row). RNAscope stainings were counterstained with DAPI. Images were acquired with the Vectra Polaris. Note that melanocytic cells show higher expression of hsa-miR-146a-5p and *MITF*, while mesenchymal cells show higher expression of *BGN*. The scale bar represents 20 μm.

**Figure 4:**
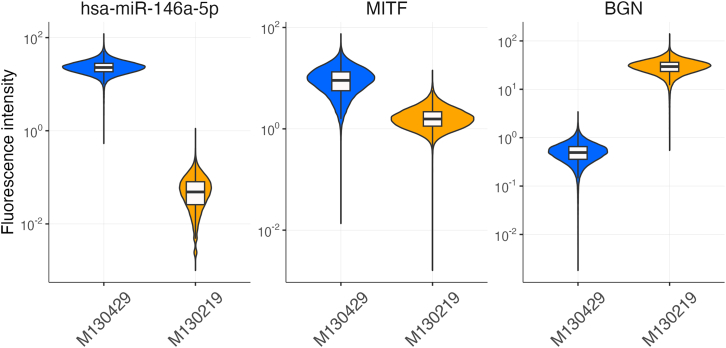
Quantitative comparison of fluorescence intensity for hsa-miR-146a-5p, MITF, and BGN in melanocytic and mesenchymal melanoma cell lines Violin plots showing the fluorescence intensity distribution of hsa-miR-146a-5p, *MITF* and *BGN* in M130429 (blue) and M130219 (orange) cell lines. Black lines represent the median and the upper and lower limit of the box depict the 25 and the 75 percentiles, respectively.

Here, we detail the outcomes of applying the RNAscope assay to two distinct melanoma cell lines: a melanocytic cell line (M130429) and a mesenchymal cell line (M130219). Both cell lines were fixed to slides and underwent pretreatment as outlined in our protocol. Following the steps for image acquisition described in the protocol, we maintained consistent acquisition times across all channels for each cell line. Our assay specifically targeted hsa-miR-146a-5p, *MITF*, and *BGN* (Figure 3). We observed variability in fluorescence intensity among the cell lines for all three RNAs. The observed miRNA and mRNAs formed large cytoplasmic clusters throughout the cells. Consequently, we quantified the signals across the entire cell area and visualized the results using a violin plot (Figure 4).

## Limitations

In the Cytospin method, cells are attached to slides through centrifugal force. Because the cells are loaded into the centrifuge as a suspension, they adopt a rounded morphology, differing from the elongated or spread-out shape typical of cells attached via adhesion. This altered cell shape reduces the cytoplasmic area.

Depending on the expression, certain miRNAs and mRNAs may not localize throughout the cytoplasm in patches. In such cases subcellular segmentation and quantification of spots may be needed.

## Troubleshooting

### Problem 1

Cells may not adhere well to the glass slide and may detach during PFA fixation or washing (Step 1b).

### Potential solution

To enhance cell attachment to slides in cases of weak adhesion, we recommend adding an extra cytospin step for cells in 4-well culture slides. Begin by removing the medium, then place the slide chambers into the Funnel Clip. Utilize the White Filter Paper to secure them firmly in position, ameliorating the setup process. Proceed to centrifuge at 800 rpm for 2 minutes. Following this, continue the protocol with the cell fixation using 4% PFA. Alternatively, chambers can be pre-coated with poly-D-lysine, poly-L-lysine, or fibronectin to enhance cell adhesion.

### Problem 2

If the signal is overly weak or excessively strong, it may indicate that the protease digestion step has not been properly optimized (Step 2).

### Potential solution

To address this issue, adjusting the protease dilution is recommended. If the signal is too weak, decrease the dilution ratio or consider using the protease in its undiluted form. Conversely, if the signal is too strong, increase the dilution ratio beyond 1:30 to achieve the desired signal intensity. Additionally, we recommend including appropriate controls to aid in troubleshooting, such as the RNAscope Plus smRNA-RNA 4-plex Positive Control Probe and the RNAscope Plus smRNA-RNA 4-plex Negative Control Probe (see details in key resources table), both available from ACD.

### Problem 3

If the signal is not uniformly distributed within the cells, appearing weaker towards the edges, this issue is likely caused by uneven distribution of the applied liquids (Step 3).

### Potential solution

To address uneven signal distribution, particularly when signals are weaker at the periphery, ensure a more balanced dispersion of liquids. Avoid drawing the hydrophobic barrier too close to the cell area, as this can reduce the available liquid volume near the barrier limiting coverage of cells with reagent.

## Resource availability

### Lead contact

For additional information, as well as requests concerning resources and reagents, please direct your inquiries to the lead contact, Mitchell P. Levesque, at Mitchellpaul.levesque@uzh.ch.

### Technical contact

Inquiries concerning the technical aspects of the protocol should be directed to the technical contact, Evelyn Lattmann, at evelyn.lattmann@usz.ch.

### Materials availability

This study did not generate new unique reagents.

### Data and code availability

The published article includes all code generated or analyzed during this study.

## Acknowledgments

This study was supported by a research grant from the Bruno Bloch Foundation. We thank Federica Sella for an introduction to the Vectra Polaris, Andelija Trajkovic for her help with visualization, and Sonja Rüegg for her advice regarding analyses.

## Author contributions

E.L. and T.G. conducted the experiments. E.L., T.G., and P.T. analyzed the data. E.L. and T.G. wrote the first manuscript draft. E.L. and M.P.L. supervised the study. All authors revised the manuscript.

## Declaration of interests

The authors declare no competing interests.
